# Everybody's Page

**Published:** 1888-04-07

**Authors:** 


### Everybody's Page.

Sleeves that tightly embrace the limbs interfere wtih the venous circulation of the part, and tend to make the fingers bluish and cold.

Freckles are due entirely to an exceptional formation of pigment in the favouring light of the sun, and sometimes fade as rapidly as they come.

Except in quite cold weather, gloves may be regarded rather as ornamental than as necessary to a perfect attire. A constant wearing of tight kid gloves must somewhat impair the circulation of the part, and render the hand attenuated by discouraging the proper use of the muscles.

Impermeable fabrics, such as macintosh cloth, do not allow of the escape of perspiration. They keep the air that is in immediate contact with the skin saturated with moisture. It is for this reason that macintosh garments are considered hot; exercise in them inducing perspiration which it does not permit to escape. This makes them very unhealthy to wear for any length of time.

The small black specks on the face, to which young people from fourteen to eighteen years of age are subject, and which are caused by obstruction of the oil glands, are easily removed by pressing with the thumb-nails or a latch-key, and so emptying the gland. When this has been done the frequent use of soap, hot water, and friction will prevent the recurrence of them to a great extent.

Soap and water and the nail-brush are all that is necessary to cleanse the nail itself. The practice of removing accumulations of dirt from beneath the free edge with a knife is not only painful and injurious to the growth, but by loosening and elevating the nail, and so increasing the excavation admits of such concretions in larger quantities. Moreover, the scratches made with the knife form little grooves, from which it is difficult to remove the foreign matter even with a brush.

In olden days long hair was looked upon as a beauty in both sexes. In the progress of Christianity, the elegance of long hair being deemed inconsistent with the profession of persons bearing the cross, it was prohibited by numerous canons and injunctions, the clergy of the Eastern Church discountenancing the practice by their own example. All such measures, however, proving ineffectual, a canon was promulgated in 1096, stating that such as wore long hair should be excommunicated while living, and remain unprayed for when dead.

Certain advances in dress reform have led to the idea that a healthy costume is of necessity an ugly costume, and that to follow in dress the laws of health is simply to obey the dictates of dowdiness. Any form of dress that is the outcome solely of certain hygienic laws is likely to be unattractive, inasmuch as it ceases to be an expression of the inner life of men and women, and becomes but the visible embodiment of certain hard scientific facts, losing that touch of individuality in which all the charm of dress consists.

With the exception of the inhabitants of Sparta and Thebes, who despised perfumes, the Greeks held ointment in great reverence. We read that it was from the summit of the sacred mountain of Thessaly that the nymph Œnone, the perfumer of Olympus, brought the secret of cosmetics. Under the rule of Solon, so great had been the abuse of cosmetics that they were made the subject of legislation, but the prohibitions of Solon were as unavailing as the satire of Socrates, who said that a slave was as good as a philosopher when he was perfumed.

The college cap, or mortar-board, while tolerated out of respect to ancient custom, is an offence against the laws of health by its weight, its uniform black hue, and its scanty provisions for ventilation.

Skilled musicians, it is said, may be able to perceive a difference between two notes of as little as 1-64th of a semitone. How they must suffer from the singing of such performers as him who sang "Deeper and Deeper Still," beginning in the key of B, going on to B flat, and ending in A, while all the time the accompaniment was being played in the key of C.

Children should not be burdened with a large amount of home study. A healthy child, from ten to fourteen years of age, who has risen at seven a.m., and has pursued a proper system of education during the day, is not in a condition to begin serious study at seven at night, or at least to do more than an hour's work. If the exigencies of modern examinations would only allow of it, the evening ought to be devoted to relaxation and pleasure.

"Eccentric," "odd" men, as the harmoniously commonplace call them, are the very salt of society. A list of the well-known ones would include some of the most cherished names in art and literature. Their heads may be "cracked," but the crack, it has been observed, lets in light. The writer of the "Castaway," and of the "Lines on Receipt of My Mother's Picture," was doubtless cracked, but the world would have been a great loser if he had been of as sound a head as the doctor who took care of him.

Very frequently a cough produces headache. The cough, which is simply an exaggerated and modified form of breathing, increases the pressure upon the great veins which bring the blood back to the heart; this increase of pressure prevents the return of the impure blood from the head, while the heart continues to pump blood thereto, and the result is a rise of pressure, which causes a sharp pain through the head with each attack of the cough.

A new vice, more dangerous even than drunkenness, is said to be gaining ground. It is the consumption of morphia, taken chiefly in the form of sub-cutaneous injections; and the evil habit seems to gain so strong a hold over those who yield to it as soon to deprive them of all self-control. However strongly they may desire to do without morphia they cannot resist the craving for it. To dipsomania may now be added morphinomania as a form of madness.

Herbert Spencer says that natural selection in marriage means not so much the choice of men as the preferences of women. The final decision rests with them; man asks, but he does not always receive. Other things being equal, women are attracted to strong men—men of indubitable power of physique, emotion, or intellect—and their instincts are to refuse the weaker specimens of the race. The average result of this feminine selection is that the best men get wives easily, while a certain proportion of the worst must remain unmarried. If Mr. Spencer's statement of facts be made into a standard of judgment, it implies a severe condemnation against all men who remain single after they can support a wife—a condemnation which those who suffer from it will resent none the less that it is presumed to be their misfortune, not their fault, that they are unmarried.

